# Intra-group competition and social dynamics regarding dispersal and maturation in wild Javan gibbon (*Hylobates moloch*)

**DOI:** 10.1038/s41598-023-34913-2

**Published:** 2023-05-22

**Authors:** Ahyun Choi, Yoonjung Yi, Ani Mardiastuti, Jae C. Choe

**Affiliations:** 1 Interdisciplinary Program of EcoCreative, Ewha Womans University, Seoul, 03760 Republic of Korea; 2 Laboratory of Animal Behaviour and Conservation, College of Biology and the Environment, Nanjing Forestry University, Nanjing, 210037 China; 3 Department of Forest Resources Conservation and Ecotourism, Faculty of Forestry and Environment, IPB University, Bogor, 16680 Indonesia; 4 Division of EcoScience, Department of Life Science, Ewha Womans University, Seoul, 03760 Republic of Korea

**Keywords:** Ecology, Behavioural ecology

## Abstract

Natal dispersal is an important life-history trait in all animal taxa. In pair-living species, parent–offspring competition derived from the offspring’s maturity can motivate the natal dispersal of offspring. However, not much has been known about the dispersal mechanisms of pair-living gibbons. To test food and mate competition as potential reasons for dispersal, we investigated the effect of the offspring age and sex on relationships between parents and offspring in wild Javan gibbon (*Hylobates moloch*) in Gunung Halimun-Salak National Park, Indonesia. We collected behavioral data for two years between 2016 and 2019. We found that aggression from parents toward offspring increased in both feeding and non-feeding context as the offspring got older. Offspring received more aggression from the parent of the same sex in the general context. While offspring decreased co-feeding and grooming time with parents as they got older, there was no change in the proximity and approach to parents. The results imply the presence of both intra-group food and mate competition which increase with the offspring’s age. We highlight that increased competition between maturing offspring and parents changes their social relationships and peripheralizes offspring from the natal group which will eventually motivate offspring to disperse in Javan gibbons.

## Introduction

Natal dispersal, the movement an animal makes from its point of origin to the place where it may reproduce, is an important life-history trait in all animal taxa^1^. Dispersal consists of three distinctive phases: emigration, movement, and immigration. Several factors influence the costs and benefits at each phase while affecting the process of dispersal^2–4^. Ultimate causes for the dispersal of animals have been suggested: to avoid inbreeding^5,6^ or to avoid resource competition^7,8^ or mate competition^9,10^. Proximate causes of dispersal are derived from intrinsic and extrinsic factors, and both influence an individual’s decision to leave natal groups^11^. While it is difficult to test the fitness of dispersal animals, proximate causes can indicate the ultimate causes of dispersal^2,12^.

The proximate causes of dispersal include individual factors (age, sex, body condition), social factors (intra-group competition, mate attraction, social bond), and ecological factors (food availability), while these factors might have entangled effects on dispersal^2,13–16^. For example, female chimpanzees (*Pan troglodytes schweinfurthii*) were more likely to disperse when there were more maternal brothers in the group^17^. Male roe deer (*Capreolus capreolus*) yearlings received more aggression from adult males as they sexually mature, which triggered them to disperse^18^. In male blue monkeys (*Cercopithecus mitis stuhlmanni*), however, weak social ties, not aggressive eviction, were important drivers for dispersal^19^. A well-fed little owl (*Athene noctua*) dispersed at a younger age than unfed ones in a poor-quality habitat and vice versa in a good-quality habitat^20^. On the other hand, spiderlings (*Amaurobius ferox*) dispersed earlier when prey lacked compared to when well-fed^21^. These results indicate that both intrinsic and extrinsic factors can shape dispersal strategies and decisions while reflecting different evolutionary causes and resulting in various consequences for population dynamics.

When it comes to pair-living mammals, the contribution of proximate factors, especially social factors, on dispersal can be different with species living in large groups due to different genetic environments since a pair-living group consists of closely related individuals, primarily of parents and offspring. In this perspective, intra-group competitions that offspring face as they grow and sexually mature can be seen as extended parent–offspring conflicts^22^. Both parents and offspring should estimate the benefits and costs of the offspring’s dispersal or their staying in the natal group. Given that offspring’s maturity and competency are obtained throughout their development, and thus older and larger offspring may pose greater competition^23^, the growth of offspring can be one of the important drivers for competitive environments in the natal group. Such parent–offspring competition can promote the dispersal of offspring while they experience changes in their relationship. In owl monkeys (*Aotus azarae*), aggression from adults toward older offspring may influence the dispersal of offspring, while mitigating intra-group competition^23^. In Japanese serow (*Capricornis crispus*), adult males were more aggressive toward male offspring than toward female offspring, indicating that competition drives offspring dispersal^24^. However, it has not been well studied what factors influence the natal dispersal of offspring in pair-living species.

Gibbons (family Hylobatidae) are well-known primates for pair-living, which can last for a long time. Both sexes disperse from their natal groups after sexual maturation (c.a., 8–10 years old^25,26^), similar to other pair-living primates such as owl monkeys^27^, tarsiers (*Tarsius spectrum*)^28^, and saki monkeys (*Pithecia aequatorialis*)^29^. Subadult gibbons, which experienced peripheralization from group members^30–32^, tended to receive aggression and were displaced by the same-sex adult^33–36^. Moreover, a captive male subadult Javan gibbon (*Hylobates moloch*) received the majority of aggression from his father when feeding or approaching the adult female^37^. These results suggest that parental aggression, probably due to food and mate competition, can trigger the natal dispersal in gibbons^9,34,38^.

However, it is difficult to understand the detailed dispersal mechanisms of wild gibbons with anecdotal descriptions from previous studies. Thus, investigating the pre-dispersal behavioral changes among group members in certain contexts would shed light on the dispersal mechanisms of wild gibbons. This study aims to investigate the effect of the offspring age and sex on social dynamics regarding intra-group food and mate competition as potential reasons for dispersal in pair-living wild Javan gibbon groups in Gunung Halimun-Salak National Park, Indonesia. Considering that older offspring may represent potential competitors both for food and mate more strongly than younger ones, we hypothesized that food competition would increase along with the offspring's age and mate competition with the interacted effects of offspring age and sex. First, for food competition, we predict that as offspring get older: (1a) they will receive more aggression from parents in the feeding context; (1b) they will decrease time spent in co-feeding with parents in the same feeding tree; (1c) they will decrease time spent in the proximity from the co-feeding parents; and (1d) they will decrease the rate of approach to feeding parents. Second, for mate competition, we predict that as offspring get older: (2a) they will receive more aggression from the same-sex parent in non-feeding contexts; (2b) they will decrease time spent in affiliative behavior (i.e., grooming) with the same-sex parent; (2c) they will decrease time spent in the proximity with the same-sex parent; and (2d) they will decrease the rate of approach to the same-sex parent in non-feeding contexts. Also, we assume that the mate competition might function regardless of the context. Thus, we additionally investigated the general agonistic relationship between Javan gibbon parents and offspring with the prediction that offspring will receive more aggression from the same-sex parent regardless of the context.

## Methods

### Study site and subject

Wild *Hylobates moloch* population in the Citalahab forest, Gunung Halimun-Salak National Park, West Java, Indonesia (S 6° 44′ 19″, E 106° 31′ 45″) has been followed since 2007, as a part of a long-term project called “Javan Gibbon Research & Conservation Project”^39–43^. We have habituated and observed two groups (A and B) since 2007 and one group (S) since 2012. We collected behavioral data from the three habituated groups during two study periods from November 2016 to August 2017 (1st period), and from December 2018 to November 2019 (2nd period), resulting in 968 and 1,242 h of observation respectively. Each group consisted of one pair of male and female adults and their offspring. When this study started in 2017, there were two offspring in each group. During the study periods, we observed six offspring in total (N_maleoffspring_ = 5; N_femaleoffspring_ = 1; Table 1) and continued to focus on them even though new offspring were born later during the study periods. There are records of the date of birth of all offspring except for one (Salwa). To estimate the age of Salwa, the inter-birth interval of this study population was calculated from offspring whose date of birth was recorded between 2010 and 2021 (N of offspring = 7; mean ± SD = 1295 ± 242 days) and subtracted that from the date of birth of the next born individual (Sanha)^44^. Even though the paternity of the study groups was not confirmed, we had not observed any adult male replacement or extra-pair copulation since 2007, which results in three reproductive cycles of adult females in group A and B, and two cycles in group S. All study offspring except Salwa were born after the groups were followed. Thus, we assume that the adult males would be the father of the offspring in each group and use the term “father” to refer putative father.

**Table 1:** Information on the identity of study subjects and ages (months) during two research periods in three wild *Hylobates moloch* groups in Gunung Halimun-Salak National Park, Indonesia.

Group	Subject offspring	Sex	Birth	Age in 1st period	Age in 2nd period
A	Amore	Male	2010–12	71–81 m.o	97–108 m.o
A	Awan	Male	2013–12	35–45 m.o	61–72 m.o
B	Kimkim	Male	2011–04	67–77 m.o	93–104 m.o
B	Komeng	Male	2014–05	30–40 m.o	57–67 m.o
S	Salwa*	Male	Unknown	72–83 m.o	99–110 m.o
S	Sanha	Female	2014–05	29–39 m.o	56–65 m.o

The asterisk indicates that the age was estimated from the inter-birth interval of this study population (mean ± SD = 1295 ± 242 days).

### Data collection

A gibbon group was followed on two consecutive days, with following of one offspring as the focal individual for the first day and the other offspring for the second day. We tried to follow all three study groups in a week. If the group of the day was found on the first day, we tried to follow the group until they entered the sleeping tree and began observation on the second day from the sleeping tree of the first day to the next sleeping tree. The orders of the focal group and the focal individual were randomly decided every week to balance the total observation hours for each study group and individual. We used the continuous focal sampling method for 15 min with 15-min breaks in between, to collect data on co-feeding, proximity, and approach^45^. Feeding behavior was considered to start when the animal put the food into his mouth and to stop when 20 s have passed without changing position or manipulating the food in the feeding tree. When the animal left the feeding tree or started eating other items, feeding on the current item was considered to have stopped. When the animal places a food item in its mouth and chews for less than one minute and stops, it was considered a casual sampling behavior instead of feeding. Co-feeding was defined as when offspring and parents were simultaneously feeding together in the same feeding tree. We recorded the identity of the adult individual who was co-feeding with the focal offspring and the total time duration of the co-feeding. We recorded the duration in proximity (less than 1 m) with an adult, the frequency of the offspring's approach toward parents within 1 m radius, and the context where the parents were (feeding or non-feeding).

Whenever the focal offspring of the observation day participated in grooming with parents (both receiving and providing), we recorded the identity of the participants and the grooming time duration. Since intra-group aggressions were not frequent, we collected direct aggressive behaviors which were clearly audible and/or visible (e.g., chasing, expelling from a tree, threatening, hitting) involving any offspring, using ad-libitum sampling. Agonistic behaviors are not different between male and female adults, and we observed both sexes of offspring being displaced or crying when they were targeted for aggression. We identified the aggressor and the receiver, and the context where the aggression occurred (feeding or non-feeding). The feeding context was defined as when any of the aggressor and the receiver was feeding before the aggression.

### Fruit availability

We estimated fruit availability based on the monthly phenology data from study groups’ feeding trees with a diameter at breast height (dbh) > 10 cm and lianas with dbh > 7 cm in 25 plots (10 m × 50 m) within their home ranges. Phenology plots were randomly selected at the crossroads of grid trails (200 m × 200 m intervals) and randomly oriented along the trail intersections^46^. We collected phenology data at the end of every month and scored the abundance of fruit with 4 levels (0: no fruits, 1: present but few, 2: moderate, 3: abundant)^46^. We calculated the fruit availability for each month by adding the scores obtained from all the trees and dividing the sum by the total number of trees.

### Statistical analysis

To test the effect of offspring age on feeding and mating competition with parents in Javan gibbons, we have four predictions in each competition hypothesis. We ran a series of Generalized Linear Mixed Models (GLMM) for all predictions (Table 2). For data analysis, we used data from days in which the observation hours exceeded 6 h (N = 229), except for aggression (model 1a, 2a) and grooming (model 2b) due to the low rate of these behaviors (N = 310).

**Table 2:** Model structure overview.

Food competition hypothesis				Mate competition hypothesis			
Model	Response	Fixed effects	Random effects	Model	Response	Fixed effects	Random effects
1a	Frequency of aggression in the feeding context	Offspring age	Gibbon group ID, subject ID	2a	Frequency of aggression in non-feeding contexts	Offspring age, sex dyad	Gibbon group ID, subject ID
1b	Co-feeding time/total feeding time	Offspring age	Gibbon group ID, subject ID	2b	Grooming time / total observation time	Offspring age, sex dyad	Gibbon group ID, subject ID
1c	Time spent within 1 m in the co-feeding context	Offspring age	Gibbon group ID, subject ID	2c	Time spent within 1 m in non-feeding contexts	Offspring age, sex dyad	Gibbon group ID, subject ID
1d	Frequency of approaching feeding parents	Offspring age	Gibbon group ID, subject ID	2d	Frequency of approaching non-feeding parents	Offspring age, sex dyad	Gibbon group ID, subject ID

#### Food competition hypothesis

We ran four GLMMs for each response variable with the monthly age of offspring as a fixed effect. Since we assume that food competition is not different depending on the sex of parents, we included the sex of parents as a control. As we focus on the effect of offspring age on food competition and fruit availability might influence food competition, we treated fruit availability as a control variable. As random intercepts, we included the focal gibbon group ID and the focal subject ID.

In model 1a, we fitted the frequency of the aggression that offspring received from parents in the feeding context as a response variable and included the observation hour as an offset term with Poisson distribution and log link function (N_withfather_ = 620; N_withmother_ = 620). In model 1b, we fitted the daily proportion of co-feeding time as a response variable with beta error distribution and logit link function. To determine the daily proportion of co-feeding time with parents, we divided the time focal offspring spent in co-feeding with a parent in the same feeding tree by the total feeding time of offspring during all 15-min focal sessions in a day (N_withfather_ = 229; N_withmother_ = 229). In model 1c, we fitted the proportion of being in proximity (< 1 m) separately to mother and father while co-feeding as a response variable with beta error distribution and logit link function. In model 1d, we fitted the frequency of the offspring’s approach to feeding parents as a response variable and included the observation hour as an offset term with negative binomial distribution and log link function.

#### Mate competition hypothesis

We ran four GLMMs for each response variable with the monthly age of offspring and parent–offspring sex dyad (same or opposite) as fixed effects. Parent–offspring sex dyad is whether the sexes of the parent and the offspring are the same or opposite. For example, the sex dyad is the same for father-son and the opposite for mother-son. Considering the effect of offspring age and sex dyad might interact, we first included the interaction as the fixed effect and if the interaction did not have a significant effect, we excluded it from the models. For the first and the second GLMM models (2a and 2b), there was no impact of the interaction, thus the interaction term was excluded in those models. As random intercepts, we included the focal gibbon group ID and the focal subject ID.

In model 2a, we fitted the frequency of the aggression that offspring received from parents in non-feeding contexts and included the observation hour as an offset term with Poisson distribution and log link function (N_same-sexdyad_ = 620; N_opposite-sexdyad_ = 620). In model 2b, we fitted the daily proportion of grooming time as a response variable with beta error distribution and logit link function. To determine the daily proportion of grooming with parents, we divided the time focal offspring spent in grooming with a parent (both receiving and providing) by the total observation time of focal offspring in a day (N_same-sexdyad_ = 310; N_opposite-sexdyad_ = 310). In model 2c, we fitted the proportion of being in proximity (< 1 m) to parents in non-feeding context as a response variable with beta error distribution and logit link function. In model 2d, we fitted the frequency of the offspring’s approach to parents in a non-feeding context as a response variable and included the observation hour as an offset term with negative binomial distribution and log link function.

To investigate the general aggression between parents and offspring, we additionally tested the effect of offspring age and the sex dyad on the aggression from parents toward offspring regardless of the context. We ran a Poisson GLMM similar to model 2a while including data from female subadult offspring Sendi in group S who had left the group before this study began. The aggression data of Sendi was collected from November 2014 to March 2016 by YY. Since her date of birth was not recorded, we used the same way as the case of Salwa to estimate the age of Sendi. Since Salwa was born after Sendi, we subtracted the inter-birth interval of this study population (mean ± SD = 1295 ± 242 days) from the estimated date of birth of Salwa^44^.

For all GLMM models, we z-transformed all covariates and checked multicollinearity using the R package *car*^47^. We did not find a problem since VIF values ranged between 1.00 and 2.00. We presented the result only when the full-null model comparison was statistically significant. All data were analyzed using R (version 4.1.0; R Core Team 2021^48^).

### Ethical note

We used data obtained only from non-invasive and behavioral observation. Our research protocol was approved by the Indonesian Ministry of Research and Technology (RISTEK), the Ministry of Forestry’s Department for the Protection and Conservation of Nature (PHKA), and the Gunung Halimun-Salak National Park (GHSNP).

## Results

### Food competition hypothesis

**Figure 1:**
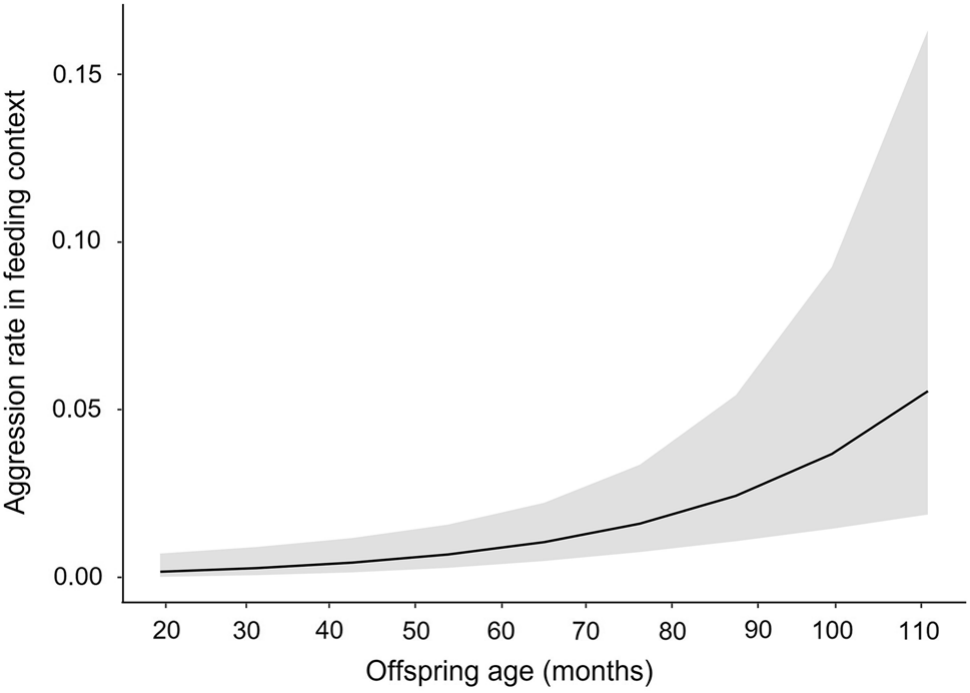
Daily aggression rate *Hylobates moloch* offspring received from parents in the feeding context in Gunung Halimun-Salak National Park, Indonesia.

**Figure 2:**
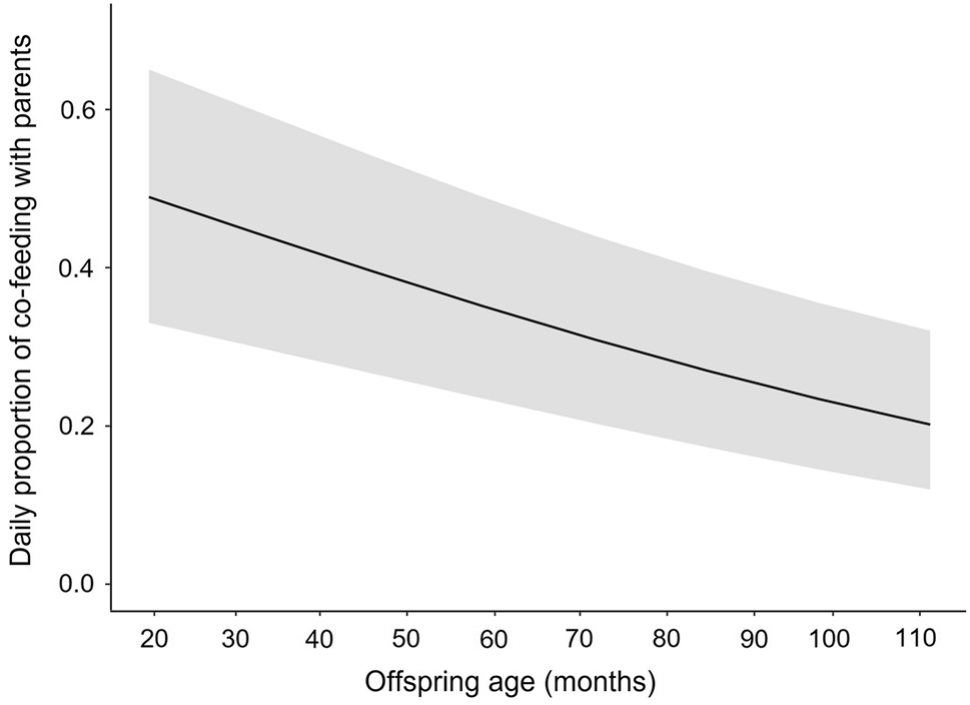
Daily proportion of co-feeding time between *Hylobates moloch* offspring with parents in Gunung Halimun-Salak National Park, Indonesia.

Javan gibbon offspring received aggression from parents in the feeding context 0.04 ± 0.22 (mean ± SD) times a day. In model 1a), the offspring age significantly affected the frequency of aggression from parents towards offspring in the feeding context (full-null model comparison: χ^2^ = 8.079, *df* = 1, *p* = 0.004; Table 3). Offspring received more aggression from parents as they got older in the feeding context (Fig. 1). In model 1b), the offspring age significantly affected the co-feeding time with parents (full-null model comparison: χ^2^ = 4.676, *df* = 1, *p* = 0.031; Table 4; Fig. 2), with offspring decreasing co-feeding time with parents as offspring get older. Model 1c and 1d, testing the effect of offspring age on proximity from parents while co-feeding (1c) and the rate of approach toward the feeding parents (1d), revealed that full models did not fit significantly better than the null models (full-null model comparison for 1c: χ^2^ = 1.999, *df* = 1, *p* = 0.157; for 1d: χ^2^ = 1.460, *df* = 1, *p* = 0.227).

**Table 3:** Effects of offspring age, sex of parent, and food availability on the frequency of aggression that *Hylobates moloch* offspring received from parents in the feeding context.

	Estimate	SE	z value	*p* value	Lower CI	Upper CI
Offspring age^1^	0.815	0.233	3.492	< 0.001***	0.357	1.272
Food availability^2^	− 0.498	0.155	− 3.221	0.001**	− 0.802	− 0.195
Parent’s sex (male)^3^	1.316	0.340	3.875	< 0.001***	0.650	1.981

^1^z-transformed (original mean ± SD: 70.797 ± 23.900).

^2^z-transformed (original mean ± SD: 0.652 ± 0.083).

^3^Dummy coded with female as the reference category.

^2,^^3^Control variables.

Significance levels: *<0.05; **<0.01; ***<0.001.

**Table 4:** Effects of offspring age, sex of parent, and food availability on the co-feeding time of *Hylobates moloch* offspring with parents.

	Estimate	SE	z value	*p* value	Lower CI	Upper CI
Offspring age^1^	− 0.383	0.091	− 4.218	< 0.001***	− 0.561	− 0.205
Food availability^2^	0.001	0.084	0.013	0.989	− 0.163	0.165
Parent’s sex (male)^3^	0.188	0.236	0.793	0.428	− 0.276	0.651

^1^z-transformed (original mean ± SD: 72.485 ± 24.125).

^2^z-transformed (original mean ± SD: 0.651 ± 0.086).

^3^Dummy coded with female as the reference category.

^2,^^3^Control variables.

Significance levels: *<0.05; **<0.01; ***<0.001.

### Mate competition hypothesis

**Figure 3:**
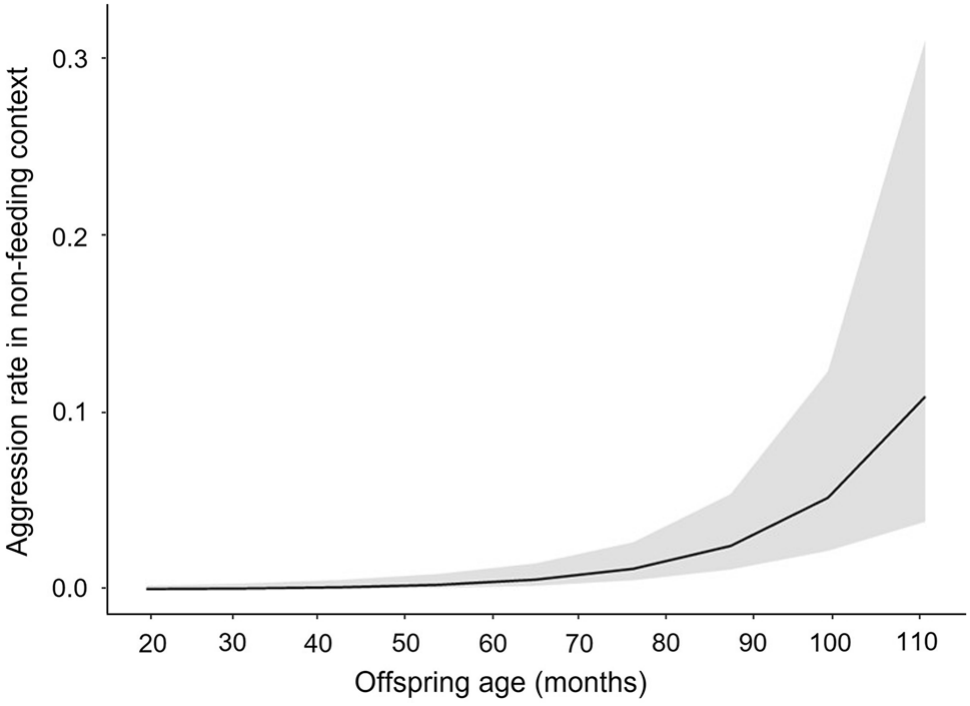
Daily aggression rate *Hylobates moloch* offspring received from parents in non-feeding contexts in Gunung Halimun-Salak National Park, Indonesia.

**Figure 4:**
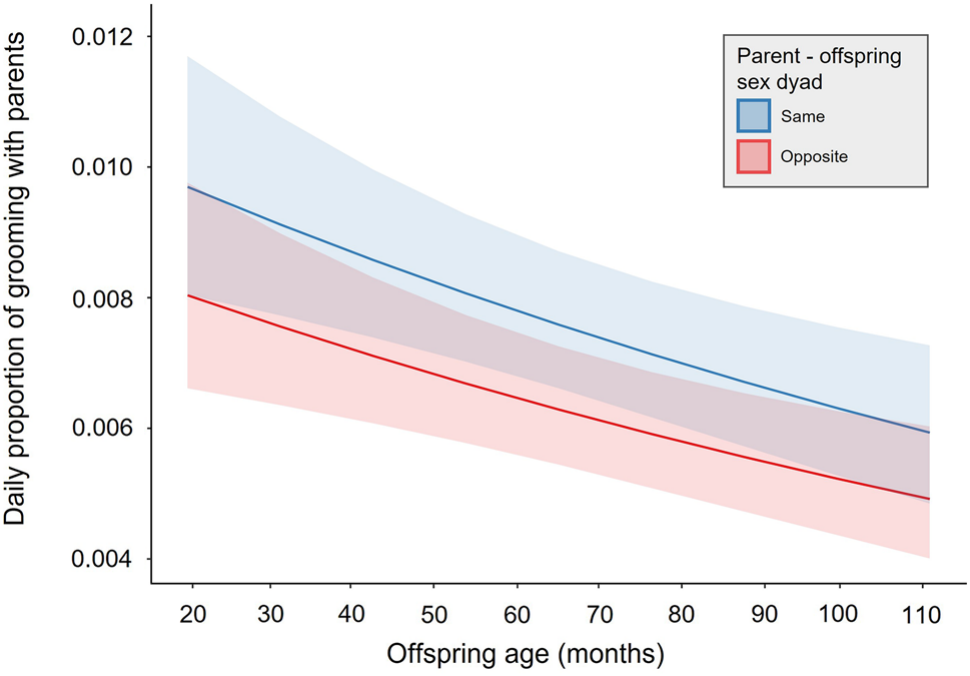
The daily proportion of grooming time between *Hylobates moloch* offspring and the parent of same-sex and opposite-sex in Gunung Halimun-Salak National Park, Indonesia.

In general, Javan gibbon offspring received aggression in non-feeding context 0.03 ± 0.20 (mean ± SD) times from the same-sex parent and 0.02 ± 0.13 (mean ± SD) times from the opposite-sex parent per day. In model 2a, offspring age significantly affected aggression from parents toward offspring in the non-feeding context, however, there was no effect of the parent–offspring sex dyad (full-null model comparison: χ^2^ = 22.902, *df* = 2, *p* < 0.001; Table 5). Javan gibbon offspring received more aggression from parents as they got older in non-feeding context (Fig. 3). In model 2b, offspring age and sex dyad had significant effects on the daily proportion of grooming with parents (full-null model comparison: χ^2^ = 9.102, *df* = 2, *p* = 0.011; Table 6). Daily grooming time between offspring and parents decreased as offspring aged (Fig. 4). However, offspring spent more time grooming with same-sex parent than with opposite-sex parent, which is opposite to our prediction. Model 2c and 2d, testing effect of offspring age, sex dyad, and the interaction on the proximity between offspring and parents (2c) and the rate of approaching toward parents in non-feeding context (2d), revealed that full models did not fit significantly better than the null models (full-null model comparison for 2c: χ^2^ = 1.331, *df* = 3, *p* = 0.722; for 2d: χ^2^ = 0.313, *df* = 3, *p* = 0.957).

**Table 5:** Effect of offspring age and parent–offspring sex dyad on the aggression that *Hylobates moloch* offspring received from parents in non-feeding context.

	Estimate	SE	z value	*p* value	Lower CI	Upper CI
Offspring age^1^	1.487	0.328	4.538	< 0.001***	0.845	2.129
Sex dyad (same)	0.647	0.372	1.737	0.82	− 0.083	1.376

^1^z-transformed (original mean ± SD = 70.797 ± 23.900).

^2^Dummy coded with opposite-sex dyad as the reference category.

Significance levels: *<0.05; **<0.01; ***<0.001.

**Table 6:** Effect of offspring age and parent–offspring sex dyad on the daily proportion of time spent in grooming between *Hylobates moloch* offspring and parents.

	Estimate	SE	z value	*p* value	Lower CI	Upper CI
Offspring age^1^	− 0.124	0.036	− 3.467	< 0.001***	− 0.194	− 0.054
Sex dyad (same)^2^	0.190	0.094	2.026	0.041*	0.008	0.372

^1^z-transformed (original mean ± SD = 71.961 ± 24.570).

^2^Dummy coded with opposite-sex dyad as the reference category.

Significance levels: *<0.05; **<0.01; ***<0.001.

**Figure 5:**
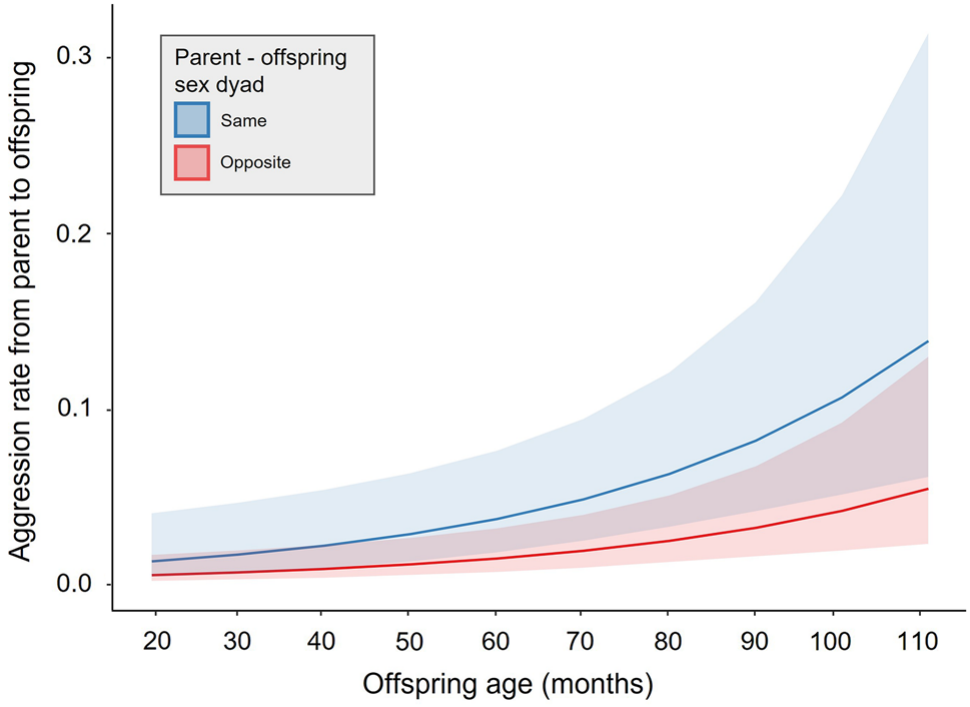
Daily aggression rate *Hylobates moloch* offspring received from the parent of the same-sex and opposite-sex in Gunung Halimun-Salak National Park, Indonesia.

We additionally tested the effect of offspring age and sex dyad between offspring and parent on aggression rate regardless of the context. We included data from one subadult female who already left the natal group (group S) before this study began. Both offspring age and sex dyad had impacts on the aggression rate that offspring received from parents (full-null model comparison: χ^2^ = 29.755, *df* = 2, *p* < 0.001; Table 7). We found that offspring received aggression more as they got older and more from the same-sex parent than from the opposite-sex parent (Fig. 5).

**Table 7:** Effects of offspring age, parent–offspring sex dyad on the aggression that *Hylobates moloch* offspring received from parents.

	Estimate	SE	z value	*p* value	Lower CI	Upper CI
Offspring age^1^	0.640	0.197	3.256	0.001**	0.255	1.026
Sex dyad (same)^2^	0.938	0.227	4.133	< 0.001***	0.493	1.383

^1^z-transformed (original mean ± SD: 74.281 ± 24.256).

^2^Dummy coded with opposite-sex dyad as the reference category.

Significance levels: *<0.05; **<0.01; ***<0.001.

## Discussion

We investigated the impact of offspring’s age on intra-group competition in a pair-living primate, highlighting potential social drivers of offspring dispersal. As Javan gibbon offspring got older, aggression received from parents increased in both feeding and non-feeding context. They received more aggression from the same-sex parent than the opposite-sex one in the general context. Javan gibbon offspring also decreased co-feeding and grooming time with parents as they got older. However, they did not change proximity and approaches toward parents both in feeding and non-feeding context. Our results partially supported increased food and mate competition with offspring’s age which may peripheralize older offspring and motivate them to disperse from their natal group.

As they got older, Javan gibbon offspring received more aggression from parents in the feeding context (prediction 1a) and decreased time spent co-feeding with parents in the same feeding trees (prediction 1b). Although we did not directly compare the feeding competency (e.g., rate of food consumption) between parents and offspring in Javan gibbons, the larger body size of growing offspring and their foraging independence can induce competition for limited food resources within the natal territory^49^. It is assumed that the home range of white-handed gibbon (*Hylobates lar*) covers an area sufficient to meet the minimum requirements of an individual or group over a set period of time^50,51^. Their territoriality with a home range that overlaps with neighboring groups may also limit the capability of expanding the home range^51^. Grown offspring’s prolonged occupancy of the limited parent’s space can be a threat to survival^52^. This can motivate parents to show aggression more frequently toward offspring in the feeding context. Offspring can also decrease co-feeding time with parents to avoid a chance to receive aggression. Our results indicate that growing offspring are gradually considered competitors for food by parents resulting in increased competition and lower tolerance between older offspring and parents. Additionally, while an offspring gets older, the group size also can increase given 3.5 years of the inter-birth interval of Javan gibbons in our study groups and the dispersal age of gibbons (c.a., 8–10 years old), which was the case in our study. This can produce more pressure on parents and older offspring because of the limited resources within the home range.

While investigating the intra-group mate competition (i.e., the relationships between offspring and the same-sex parent in non-feeding contexts) in Javan gibbons, we found that Javan gibbon offspring received more aggression from parents in non-feeding contexts as they got older. Although they did not receive aggression especially more from the same-sex parent in non-feeding contexts, we found that Javan gibbon offspring received more aggression from the same-sex parent in general without considering the specific context. This result showed the same pattern as the descriptions from previous studies that parents become aggressive toward maturing offspring of the same sex in other gibbon species, white-handed gibbons^53^, siamangs (*Symphalangus syndactylus*)^54^, and western black crested gibbons (*Nomascus concolor*)^36^. It has been suggested that gibbons reach sexual maturity at the age of about six to eight years^25,55,56^. As offspring reach sexual maturity, they can challenge the breeding position in the natal group^57,58^. Even in pair-living species, intrasexual competition between offspring and the parent of the same sex can occur depending on the demographic changes (i.e. replacement of opposite-sex parent), characteristics of the floater population, or chances for extra-pair copulation^34,59–61^. Thus, Javan gibbon parents may also increase aggression toward the same-sex offspring to exclude potential reproductive competitors.

Javan gibbon offspring reduced time spent in grooming with parents as they got older. This implies weaker social bonds between older offspring and parents, which have been observed in other dispersing animals with group members^19,62^. However, offspring generally spent time in grooming more with the same-sex parent than with the opposite-sex parent throughout their age. We suggest alternative explanations for this contradictory result. First, most of our study offspring were male (N_maleoffspring_ = 5; N_femaleoffspring_ = 1), and the age of the only female offspring was covered from 29 to 65 months during the research period, which did not involve her subadult stage. This may produce biased results toward males when investigating the effect of sex on relationships. Second, indirect paternal care of adult male Javan gibbons may enhance the biased effect of sex on the relationship between offspring and parents. Even though fathers of most gibbon species except for the siamangs do not participate in direct parental care for infants (i.e., carrying infants), they can provide paternal care in other ways by defending, grooming, and playing, especially after the offspring’s weaning from the mother^63,64^. Thus, while increased aggression rate in non-feeding contexts might reflect the higher competition between offspring and parents in general, mate competition between male offspring and fathers might show more evident effects later than 9.2 years old of age which our study covered.

Aggression from natal group members can drive individuals to disperse from the group^18,65^, which is the case for offspring and parents in monogamous species^23,24^. Even though the within-group aggressive behaviors are not frequent in gibbons, we revealed that the aggression from parents to their offspring increased throughout the prolonged period of the maturation in Javan gibbons. At the end of the study period, we had three focal offspring that entered the subadult age class, and two of them left their natal group after the study period ended. Even though we could not collect data until their actual dispersals, we suggest that gradually increased aggression from parents toward older offspring may represent the increased parent–offspring competition caused by the maturation of the offspring and eventually contribute to their natal dispersal. Aggression from parents also may make offspring avoid co-feeding or decrease affiliative behaviors with parents. Peripheralization of offspring is a prolonged and subtle process over many years and influences the decision on the dispersal of offspring in Javan gibbons.

The spatial proximity through approaching adults often goes with increasing competition resulting in more opportunities for aggression^66^. Individuals may reduce spatial proximity and decrease their approach to other individuals to avoid competition. However, our results did not confirm this among Javan gibbon parents and offspring in both feeding and non-feeding context along with the offspring’s age. Javan gibbon offspring did not change time spent in proximity and approaching rate to parents throughout their development (rejection of prediction 1c, 1d, 2c, and 2d). While having high coordination of the movement among group members, Javan gibbon offspring rarely spent time at a close distance (i.e., within a radius of 1 m) from parents after the juvenile period. Even subadult offspring often kept their distance far from the other group members until we could not visually check their locations. In wild siamangs, infants already spent most of their time > 2.5 m from their mothers after 16 months old^67^. The criterion of proximity as 1 m in this study may not be proper to investigate changes in the relationship shown in the inter-individual distance for grown-up individuals in wild gibbons. We, therefore, suggest investigating inter-individual distance and ranging behaviors to study further the relationships reflected in spatial behavior in Javan gibbons.

In our study groups, two male subadults from groups B and S disappeared and never have been observed again after the research period ended. They were nine and ten years old respectively, and we assume that they dispersed from the natal group. This was within the known age of dispersal of other gibbon species (white-handed gibbons: 8 – 10 years old^25^; western black crested gibbons: 8 – 12 years old^26^). Javan gibbon offspring left their natal group about two years after they got sexual maturity, which suggests that they delayed dispersal as reported in wild white-handed gibbons^25^. Delayed dispersal can be advantageous in a saturated habitat with limited opportunities to find vacant space^25^. This might be one of the reasons for the delayed dispersal of the subadult offspring in our study groups. Their natal territories are surrounded by village and human-made roads on one side and by neighboring groups on the other side with overlapped territories. Besides the ecological causes, delayed dispersal of offspring may benefit parents by aiding resource defense from outgroups or caring siblings through social grooming or playing, thus the parents show tolerance toward subadult offspring^25,37^. While delaying their reproduction in the natal group, mature offspring may find opportunities to take over the natal territory or establish their own territory near the natal territory^25,34,68^. This can be the case for one male subadult (Amore) from group A, who was observed to stay in the natal group until he became 12 years old (unpublished data). We have observed that all subadult offspring except this individual in our study groups left their natal groups since 2007 when the project began. Further observations in the future will allow us to understand how long the young gibbon delays dispersal or how he establishes his territory.

Natal dispersal is an important milestone during the life history of an individual, and it is related to not only individual fitness but also other important traits such as social and genetic structure, and population dynamics^69,70^. Different from animals living in multi-female/male groups usually with sex-biased dispersal, intra-group competition and its role in the bisexual dispersal of offspring have been underestimated and not been documented very well in pair-living species such as gibbons. Our study highlights that increased competition between subadult offspring and parents changes social dynamics and peripheralizes offspring which would eventually make them leave their natal groups. While our study focused on social dynamics influenced by the maturation of offspring, further studies can consider other ecological factors for a more comprehensive discussion on parent–offspring competition as a dispersal mechanism. Further studies also should capture the process of dispersal and the genetic consequences on the population. Our findings offered an understanding of social dynamics in relation to the dispersal of wild Javan gibbons, which will contribute to extending our knowledge of the evolution of dispersal and social system.

## Data Availability

Data supporting the results and conclusions of this study can be available from the corresponding author upon request.
